# Effects of 8 weeks of combined strength and plyometric training on lower limb vertical stiffness and jump performance in elite long jump athletes

**DOI:** 10.3389/fphys.2025.1692254

**Published:** 2025-11-10

**Authors:** Zhanming Xu, Jiawei Sun, Jianing Gu, Laikang Yu

**Affiliations:** 1 College of Education, Beijing Sport University, Beijing, China; 2 Key Laboratory of Sport Training of General Administration of Sport of China, General Administration of Sport, Beijing, China; 3 Beijing Key Laboratory of Sports Performance and Skill Assessment, Beijing Sport University, Beijing, China; 4 Department of Strength and Conditioning Assessment and Monitoring, Beijing Sport University, Beijing, China

**Keywords:** strength training, plyometric training, vertical stiffness, elastic energy utilization, jump performance, long jump

## Abstract

**Background:**

The approach run in the long jump relies heavily on lower limb vertical stiffness and elastic energy utilization (EEU). While conventional strength training enhances maximal force, it may not adequately improve stiffness or stretch-shortening cycle efficiency. Plyometric training (PT), by contrast, specifically targets these qualities. This study examined whether combining strength and plyometric training yields superior neuromuscular adaptations in elite long jump athletes.

**Methods:**

Twenty-four elite long jump athletes (12 male, 12 female) were allocated to a strength training group (ST) or a combined strength plus plyometric training group (ST + PT). Both groups trained twice weekly for 8 weeks, with ST loads set at 80%–85% one-repetition maximum (1RM). Outcome measures included 1RM back squat strength, countermovement jump (CMJ) height, vertical stiffness (Kvert, bilateral and unilateral), and EEU.

**Results:**

Both the ST and ST + PT interventions significantly improved 1RM strength in elite long jump athletes (P < 0.001). However, significant enhancements in vertical stiffness (Kvert-L, Kvert-R, Kvert-B; P < 0.01), EEU (P < 0.05), and CMJ height (P < 0.01) were observed exclusively in the ST + PT group. Furthermore, male athletes demonstrated greater training-induced adaptations than females, particularly in 1RM (P < 0.001), vertical stiffness (Kvert-R, P = 0.010; Kvert-B, P = 0.001), and CMJ height (P = 0.003).

**Conclusion:**

An 8-week program combining strength and plyometric training is more effective than strength training alone for enhancing lower-limb stiffness, EEU, and jump performance in elite long jump athletes, particularly in males. These findings support integrating plyometric modalities into pre-competition training cycles.

## 1 Introduction

A successful long jump takeoff depends critically on the approach run, whose primary goal is to achieve maximal controllable horizontal velocity. Previous research has identified phases of the approach: acceleration, maximal velocity, and velocity maintenance (Hossain et al., 2024). Long jump performance is strongly correlated with maximal approach velocity and sprint ability, with elite jumpers demonstrating world-class sprinting capacity. Running technique, leg strength, lower limb stiffness, and elastic energy utilization (EEU) are key determinants of approach velocity. The coordinated interaction among these factors facilitates the efficient transformation of muscular and elastic energy into horizontal speed.

The stretch–shortening cycle (SSC) underpins explosive lower limb performance. It involves eccentric muscle action with tendon elongation followed by concentric contraction and tendon recoil (Pedley et al., 2022). SSC efficiency is enhanced through neural, muscular, and musculotendinous adaptations, including improved motor unit recruitment, greater tendon stiffness, and increased rate of force development (RFD) (Waugh et al., 2012; Radnor et al., 2018). These adaptations improve force production and energy reutilization, thereby supporting sprinting and jumping performance (Radnor et al., 2018; Tumkur Anil Kumar et al., 2021).

Lower limb muscle strength and power are fundamental for jumping, which relies on maximal voluntary contraction (MVC) and SSC function (Newton et al., 2006; Riggs and Sheppard, 2009; Wang et al., 2015). Plyometric training (PT) is widely employed to enhance these qualities, with vertical jump height often used as an outcome measure. Kinetic analyses of adult basketball and elite beach volleyball athletes show that joint moments, peak power, and RFD are strongly associated with jump performance (Gerodimos et al., 2008; Riggs and Sheppard, 2009). More recently, stiffness has emerged as a key variable for understanding lower limb function during explosive movements (Yoon et al., 2007; Brughelli and Cronin, 2008a; Maloney and Fletcher, 2021; Zhang et al., 2023).

Evidence indicates that optimizing SSC function enhances both vertical leg stiffness and EEU (Satkunskiene et al., 2021). PT improves elastic energy storage and release, augments stretch-reflex contribution, increases neuromuscular recruitment, and reduces interlimb asymmetries, thereby supporting more balanced and efficient movement patterns (Flanagan and Comyns, 2008; Radnor et al., 2018; Bettariga et al., 2023; De Maio et al., 2023). Systematic training interventions, particularly strength and PT, have been shown to enhance EEU by increasing maximal absolute strength, RFD, eccentric strength, SSC efficiency, and lower limb stiffness (Chimera et al., 2004). Plyometric exercises such as depth jumps, bounding, and hopping exploit musculotendinous elastic properties, thereby facilitating energy storage during eccentric loading and release during concentric contraction.

EEU refers to the ability of the muscle-tendon unit to store and reuse elastic energy during SSC movements, contributing to enhanced explosive performance (Harry et al., 2019). EEU is influenced by several factors, including the magnitude and velocity of muscle stretch, the stiffness and activation state of the musculotendinous unit (MTU), muscle length at the end of eccentric contraction, and the duration of the amortization phase. Functionally, this mechanism is integral to running biomechanics, in which muscles serve not only as force- and power-generating engines, but also dampers, springs, and stabilizers (Lai et al., 2019). Accordingly, maximal strength, eccentric capacity, SSC function, and stiffness collectively govern the efficiency of EEU. Running, the key component of the long jump approach, is characterized by repeated cycles of eccentric and concentric contractions in which muscles, tendons, and ligaments operate as spring-like systems to maximize elastic energy reutilization. Empirical evidence suggests that elastic energy reuse may account for 30%–40% of the total energy expenditure in running, with contributions exceeding 50% during sprinting (Støren et al., 2008). These findings underscore the relevance of PT as an intervention for enhancing EEU. Indeed, PT has been shown to improve lower limb muscle strength and stiffness in male volleyball players following 6 weeks of training (Mroczek et al., 2019).

Furthermore, combining strength and plyometric training yields superior improvements in strength and power compared with either modality alone, with consistent benefits reported in male and female athletes across various sports, including soccer, basketball, volleyball, and handball (Dell’Antonio et al., 2022; Jakšić et al., 2023; Huang et al., 2024; Norgeot and Fouré, 2024). However, most studies have focused on endurance running or general athletic populations, and the mechanisms underlying explosive performance in specialized jumping events, such as the long jump approach, remain poorly understood. In particular, while the roles of SSC, stiffness, and EEU have been explored in other populations, it is unclear how these factors interact to influence approach velocity and jump performance in elite long jump athletes.

Therefore, this study investigates the effects of an 8-week combined strength and plyometric training program on lower limb vertical stiffness (Kvert-B, Kvert-L, Kvert-R) and vertical jump performance in elite long jump athletes, compared with conventional resistance training. We hypothesize that combined strength and plyometric training will significantly enhance vertical stiffness, EEU, and vertical jump height, providing practical insights for specialized long jump training.

## 2 Materials and methods

### 2.1 Participants

Sample size was estimated using G*Power software (repeated-measures ANOVA, d = 0.25, α = 0.05, power = 0.80), indicating a minimum requirement of 24 participants. Accordingly, 24 elite long jump athletes (12 males and 12 females; 21 left-leg dominant, 3 right-leg dominant) from Beijing Sport University were recruited and randomly assigned to either a conventional strength training group (ST, *n* = 12, 6 males and 6 females) or a combined ST and PT group (ST + PT, *n* = 12, 6 males and 6 females). Inclusion criteria required participants to have achieved at least the performance standard of a Chinese National Level I athlete in the long jump event, to be free from any cardiovascular or respiratory disorders, to have had no lower-limb joint injuries or neurological or orthopedic conditions affecting the lower extremities within the previous 6 months, to be in good physical condition at the time of assessment, and to have demonstrable prior experience with squat training. All participants volunteered during the off-season and provided written informed consent after being fully informed of the study purpose, procedures, risks, benefits, and potential discomforts. Descriptive characteristics for the participants can be viewed in Table 1. Baseline characteristics did not differ significantly between groups (P > 0.05). The study protocol was approved by the Ethics Committee of Beijing Sport University (Approval number: 2025266H), and all procedures were carried out in accordance with the Declaration of Helsinki.

**TABLE 1:** The descriptive characteristics of the participants.

Group	Sex	Age (years)	Height (cm)	Body weight (kg)	Training experience (years)
ST (*n* = 12)	Male (*n* = 6)	19.38 ± 1.06	182.44 ± 6.17	70.30 ± 8.74	8.72 ± 2.01
	Female (*n* = 6)	20.75 ± 2.63	170.25 ± 4.86	57.58 ± 5.90	8.37 ± 1.47
ST + PT (*n* = 12)	Male (*n* = 6)	20.33 ± 2.34	183.60 ± 4.34	70.03 ± 5.14	9.02 ± 1.88
	Female (*n* = 6)	19.50 ± 2.43	171.33 ± 5.39	58.13 ± 5.35	8.94 ± 1.72

### 2.2 Study design

This study employed a randomized controlled design with repeated measures. Following baseline assessments, participants underwent an 8-week intervention of either ST alone or ST combined with PT (ST + PT). All training sessions were conducted under the supervision of qualified coaches, and participants were familiarized with all procedures prior to data collection. In addition, all participants underwent 1 week of familiarization with the training program before the commencement of formal training. To ensure consistency, all tests and training sessions were performed on the same indoor track surface, participants wore standardized athletic footwear, and testing was conducted at similar times of day under comparable environmental conditions (e.g., temperature and humidity).

### 2.3 Training program

Participants were required to demonstrate proper lifting technique and complete familiarization with all assessment procedures prior to the intervention to minimize errors arising from technical or procedural inexperience. Each training session commenced with a standardized warm-up consisting of 5 min of jogging, muscle activation, dynamic stretching, and neuromuscular activation exercises (Table 2). While maintaining their habitual endurance training, participants performed two supervised strength-training sessions per week, with all loads prescribed at 80%–85% of each individual’s one-repetition maximum (1RM).

**TABLE 2:** Warm-up programs for ST and ST + PT protocols.

Warm-up component	ST	ST + PT
Jogging	5 min	5 min
Muscle activation	Shoulder circles: 1 set × 12 reps Mountain climber: 1 set × 18 reps Single leg glute bridges: 1 set × 12 reps Superman: 1 set × 8 reps Squat: 1 set × 8 reps	Shoulder circles: 1 set × 12 reps Mountain climber: 1 set × 18 reps Single leg glute bridges: 1 set × 12 reps Superman: 1 set × 8 reps Squat: 1 set × 8 reps
Dynamic stretching	Inchworm: 1 set × 8 reps 90/90 for hips: 1 set × 8 reps Lunge walk: 1 set × 8 reps Walking knee lift: 1 set × 8 reps	Inchworm: 1 set × 8 reps 90/90 for hips: 1 set × 8 reps Lunge walk: 1 set × 8 reps Walking knee lift: 1 set × 8 reps
Neuromuscular activation	Split squat jump: 1 set × 5 reps Squat jump: 1 set × 5 reps Box jump: 1 set × 5 reps	Split squat jump: 1 set × 5 reps Squat jump: 1 set × 5 reps Box jump: 1 set × 5 reps

The training program was designed based on previous studies (Papla et al., 2023; Osses-Rivera et al., 2024). As detailed in Table 3, in the ST + PT group, three complex-training pairs were performed sequentially: (1) barbell squats followed by 40-cm box depth jumps; (2) split squats followed by double-leg 40-cm hurdle jumps; and (3) seated unilateral calf raises followed by single-leg jumps. Intra-complex and inter-set rest intervals were standardized at 4 min. The ST group performed the same strength exercises (barbell squats, split squats, and seated unilateral calf raises) at 80%–85% 1RM for three sets each, with 3-min inter-set rest. To equate total training volume, the ST group completed two additional sets per exercise using the same intensity and repetition scheme to match the plyometric workload of the ST + PT group.

**TABLE 3:** Training schedule of ST and ST + PT protocols.

Group	Exercise	Intensity	Sets	Repetitions	Rest interval (min)
ST (*n* = 12)	Knee 90° squat	80%–85% 1RM	5	5	3
	Split squat	80%–85% 1RM	5	5	3
	Single-leg seated calf raise	80%–85% 1RM	5	5/side	3
ST + PT (*n* = 12)	Knee 90° squat	80%–85% 1RM	3	5	4
	Box jump	40 cm height	3	6	
	Split squat	80%–85% 1RM	3	5	4
	Double-leg hurdle jump	40 cm hurdle	3	6	
	Single-leg seated calf raise	80%–85% 1RM	3	5/side	4
	Single-leg jump	Body weight	3	6/side	

Both training protocols were implemented over 8 weeks, with the ST + PT program structured into three progressive phases: (1) a 2-week adaptation phase at moderate intensity, (2) a 2-week enhancement phase at moderate-to-high intensity, and (3) a 4-week consolidation phase at high intensity. Training progression and exercise execution were continuously supervised by the same coach, who monitored jump height, velocity, and technical quality throughout each plyometric session.

### 2.4 Measurements

#### 2.4.1 Anthropometrics

Stature was assessed barefoot with a wall-mounted stadiometer (Model H-630; Marsden Weighing Group, London, United Kingdom) read to the nearest 0.1 cm; body weight was obtained on a calibrated digital scale. To standardize hydration and neuromuscular status, participants abstained from alcohol and caffeine, refrained from strenuous exercise, and recorded ≥8 h of sleep during the 24 h preceding all measurements.

#### 2.4.2 One-repetition maximum (1RM)

1RM back-squat testing was executed using validated procedures (Grgic et al., 2020). Participants first completed a 15-min warm-up consisting of dynamic stretching and body-weight exercises to optimize joint range of motion and neuromuscular activation. Thereafter, four incremental warm-up sets were performed: 10 repetitions at 50%, 5 at 70%, 3 at 80%, and 1 at 90% of the individually estimated 1RM, derived from age, body mass, training history, and coach recommendations. Subsequently, participants performed three to four maximal attempts to establish actual 1RM, separated by 3–5 min of passive recovery. Technical criteria required full-depth squats with the femur at least parallel to the floor at the lowest position, while two certified spotters provided continuous safety supervision throughout testing.

#### 2.4.3 Countermovement jump (CMJ)

CMJ testing consisted of three consecutive trials separated by 10-s passive recovery intervals (Yuan et al., 2023). For each trial, participants assumed an upright stance with hands akimbo and feet positioned at approximately shoulder width. Upon the verbal cue “Jump”, they executed a self-selected-depth countermovement followed by an explosive vertical take-off. During flight, knees remained fully extended and hands remained on hips. The trial yielding the highest jump height was retained for analysis. Ground-reaction forces were recorded via a three-dimensional force platform (Kistler 9281CA, Switzerland) at 1,000 Hz. Trials were repeated if protocol deviations occurred, and the platform centre was delineated with athletic tape to standardize foot placement.

#### 2.4.4 Vertical stiffness (Kvert)

Lower limb vertical stiffness was assessed using the 5-jump test on a three-dimensional force platform (Kistler 9281CA, Switzerland; sampling frequency: 1,000 Hz). After a standardized 5-min warm-up, participants performed five consecutive maximal vertical jumps on a force platform, ensuring minimal ground contact time and minimal knee flexion (straight legs). Prior to formal data collection, participants completed standardized familiarization trials to ensure procedural competency.

Given that long jump is an asymmetrical sport, with athletes generating and transferring horizontal velocity differently between legs during the approach and take-off phases, both bilateral and unilateral stiffness assessments were conducted. This approach allows for the evaluation of potential side-to-side differences in lower-limb function and their contribution to maintaining approach velocity and optimizing jump performance.

Following an adequate recovery period, each participant executed two maximal-effort test sequences under both bilateral and unilateral conditions, separated by a 2-min passive rest interval. For data processing, the contact times (TC) of the middle three consecutive hops within each trial were averaged (Morin et al., 2005). Vertical stiffness (Kvert) was subsequently computed via the following equation, and the highest Kvert value obtained across all valid trials were retained for statistical analysis (Equations 1, 2).

$$Kvert=\frac{F\max}{\Delta y}$$ (1)

$$\Delta y=\frac{F\max\times Tc^{2}}{m\Pi^{2}}+\frac{gTc^{2}}{8}$$ (2)

Note: *F*_max_ is the peak vertical ground reaction force; *Δy* is the vertical displacement of the center of mass; *m* is the body mass; *Tc* is the ground contact time, determined using a 10 N threshold on the force plate signal; *g* is the acceleration due to gravity, set at 9.8 m/s^2^.

#### 2.4.5 Elastic energy utilization (EEU)

EEU was assessed immediately after the CMJ protocol. Upon completion and an adequate recovery period, participants performed squat-jump (SJ) trials on the identical Kistler 9281CA triaxial force platform (1,000 Hz). After calibration, body-mass recording and tare-zeroing, the sequence proceeded as follows: (1) on the “ready” command, subjects adopted an upright stance, hands on hips, gaze forward; (2) upon “start”, they descended to a semi-squat position with the knee angle verified at 90° (manual goniometer, minimal intrusion); (3) after angle confirmation, a maximal vertical jump was executed, landing on the platform and re-establishing balance; (4) force-time data were continuously acquired. Two familiarization jumps were followed by three recorded trials separated by 60 s; the trial yielding the greatest jump height was retained. EEU was subsequently computed from the CMJ and SJ outcomes using the established formula (Equation 3).

$$EEU=\frac{\left(Hcmj-Hsj\right)}{Hcmj}\times 100\%$$ (3)

### 2.5 Statistical analysis

Data were presented as mean ± standard deviation (SD). Normality was assessed using the Shapiro-Wilk test. A three-way repeated measures ANOVA (group × gender × time) was employed to evaluate training-related changes in 1RM, vertical stiffness, EEU, and jump performance. Mauchly’s test was used to assess the assumption of sphericity, and Greenhouse-Geisser corrections were applied when the sphericity assumption was violated. To control for the increased risk of Type I error due to multiple comparisons, post-hoc pairwise comparisons were conducted using Bonferroni adjustment. Effect sizes were calculated using Hedges’ g and interpreted as small (0.2), medium (0.5), or large (0.8) (Fritz et al., 2012). Statistical significance was set at P < 0.05. All analyses were performed using SPSS 23.0 (IBM, United States).

## 3 Results

### 3.1 Effects of combined strength and plyometric training on lower limb maximal strength

Three-way repeated measures ANOVA revealed an interaction between group, gender, and time (F_(1,23)_ = 0.858, P = 0.033). Between-subjects contrasts indicated a non-significant group effect (F_(1,23)_ = 2.132, P = 0.152), a highly significant gender effect (F_(1,23)_ = 53.257, P < 0.001), and a non-significant group × gender interaction (F_(1,23)_ = 1.086, P = 0.304). As shown in Table 4, post-intervention, both the ST and ST + PT groups demonstrated significant increases in 1RM for males (both P < 0.001) and females (both P < 0.001). No between-group differences emerged, and males exhibited significantly greater absolute 1RM values than females (P < 0.001). Hedges’ g for between-group comparisons post-intervention were −1.192 (95% confidence interval [CI], −27.48 to 7.96; P = 0.248) for males and 0.179 (95% CI, −20.07 to 21.49; P = 0.941) for females.

**TABLE 4:** The assessment results for ST group and ST + PT group before and after the 8-week training.

Variable	Gender	ST (*n* = 12)		ST + PT (*n* = 12)		The effects of group, gender, time, and their interactions (P)			
		Pre	Post	Pre	Post	Group	Gender	Group × gender	Group × gender × time
1RM squat strength (kg)	Male	100.00 ± 12.72	118.57 ± 15.85^a^	111.67 ± 12.39	128.33 ± 11.30^a^	0.152	<0.001	0.304	0.858
	Female	75.00 ± 11.63	90.00 ± 12.39^a^	79.29 ± 20.20	89.29 ± 19.18^a^				
Kvert–L (KN·m^-1^)	Male	12.91 ± 1.58	14.09 ± 1.35^b^	12.76 ± 1.08	14.00 ± 1.63^c^	0.73	0.002	0.922	0.219
	Female	11.67 ± 2.21	12.28 ± 1.02	10.22 ± 1.23	13.30 ± 2.57^c^				
Kvert–R (KN·m^-1^)	Male	13.04 ± 1.73	13.65 ± 1.37	11.66 ± 2.16	12.72 ± 2.16^c^	0.071	<0.001	0.709	0.908
	Female	10.9 ± 2.47	11.48 ± 1.57	9.79 ± 1.10	11.06 ± 1.29^c^				
Kvert–B (KN·m^-1^)	Male	26.39 ± 3.64	28.22 ± 1.35	27.87 ± 6.36	30.41 ± 6.68^b^	0.362	<0.001	0.604	0.873
	Female	19.63 ± 3.07	21.46 ± 3.09^b^	20.19 ± 4.19	21.91 ± 4.15^c^				
EEU (%)	Male	9.31 ± 3.25	9.48 ± 3.7	7.87 ± 1.97	8.48 ± 2.35^b^	0.552	0.609	0.026	0.892
	Female	7.35 ± 1.98	7.41 ± 1.97	9.01 ± 1.77	9.90 ± 2.01^b^				
CMJ (cm)	Male	38.59 ± 4.12	41.13 ± 5.22	39.77 ± 2.71	42.00 ± 2.42^a^	0.998	<0.001	0.350	0.809
	Female	35.12 ± 4.36	36.52 ± 4.32	33.73 ± 2.37	35.87 ± 3.34^c^				

^a^ RM, one-repetition maximum; EEU, elastic energy utilization; CMJ, countermovement jump.

^b^ Significantly different between pre-training and post-training, P < 0.001.

^c^ Significantly different between pre-training and post-training, P < 0.05.

^d^ Significantly different between pre-training and post-training, P < 0.01.

### 3.2 Effects of combined strength and plyometric training on lower limb vertical stiffness

Three-way repeated measures ANOVA revealed no interaction between group, gender, and time in Kvert-L (F_(1,23)_ = 1.561, P = 0.219), Kvert-R (F_(1,23)_ = 0.013, P = 0.908), and Kvert-B (F_(1,23)_ = 0.026, P = 0.873). Between-subjects effects revealed non-significant group effects (Kvert-L, F_(1,23)_ = 0.121, P = 0.730; Kvert-R, F_(1,23)_ = 3.435, P = 0.071; Kvert-B, F_(1,23)_ = 0.85, P = 0.362), significant gender effects (Kvert-L: F_(1,23)_ = 10.633, P = 0.002; Kvert-R: F_(1,23)_ = 14.319, P < 0.001; Kvert-B: F_(1,23)_ = 34.346, P < 0.001), and non-significant group × gender interaction for Kvert-R (F_(1,23)_ = 5.344, P = 0.709). As shown in Table 4, post-intervention, the ST + PT group exhibited significant improvements in all stiffness measures for both sexes (males: Kvert-L, P = 0.007; Kvert-R, P = 0.004; Kvert-B, P = 0.003; females: Kvert-L, P = 0.007; Kvert-R, P = 0.007; Kvert-B, P = 0.009). No between-group differences were observed (all p > 0.05). Males displayed significantly greater Kvert-R (P = 0.010) and Kvert-B (P = 0.001) than females. Post-intervention between-group effect sizes were as follows: Kvert-L (males: Hedges’ g = 0.178, P = 0.919, 95% CI, −1.84 to 2.02; females: Hedges’ g = −0.567, P = 0.389, 95% CI, −3.55 to 1.51); Kvert-R (males: Hedges’ g = 1.010, P = 0.395, 95% CI, −1.40 to 3.26; females: Hedges’ g = 0.724, P = 0.624, 95% CI, −1.43–2.27); and Kvert-B (males: Hedges’ g = −0.947, P = 0.466, 95% CI, −9.18 to 4.81; females: Hedges’ g = −0.418, P = 0.836, 95% CI, −5.16 to 4.26).

### 3.3 Effects of combined strength and plyometric training on lower limb EEU

Three-way repeated measures ANOVA revealed an interaction between group, gender, and time (F_(1,23)_ = 0.019, P = 0.892). Between-subject contrasts further revealed neither a main effect of group (F_(1,23)_ = 0.360, P = 0.552) nor of gender (F_(1,23)_ = 0.266, P = 0.609); nevertheless, a significant group × gender interaction emerged (F_(1,23)_ = 5.344, P = 0.026). As shown in Table 4, post-intervention, EEU did not change in the ST group (males: P = 0.612; females: P = 0.880), whereas the ST + PT group exhibited significant improvements (males: P = 0.023; females: P = 0.012). Between-group Hedges’ g values were 0.775 (95% CI, −2.99 to 4.99; P = 0.589) for males and −1.635 (95% CI, −5.05 to 0.07; P = 0.056) for females.

### 3.4 Effects of combined strength and plyometric training on lower limb jump performance

Three-way repeated measures ANOVA revealed no interaction between group, gender, and time (F_(1,23)_ = 0.059, P = 0.809). Between-subjects effects revealed non-significant group (F_(1,23)_ = 0.000, P = 0.998) and group × gender (F_(1,23)_ = 0.895, P = 0.350) effects, and a highly significant gender effect (F_(1,23)_ = 21.94, P < 0.001). As shown in Table 4, post-intervention, the ST + PT group demonstrated significant improvements in CMJ height (males: P < 0.001; females: P = 0.003), whereas between-group differences were non-significant. Males exhibited significantly greater CMJ height than females (P = 0.003). Hedges’ g values were −5.670 (95% CI, −6.42 to 4.68; P = 0.722) for males and 0.485 (95% CI, −4.32 to 5.62; P = 0.777) for females.

## 4 Discussion

### 4.1 Combined strength and plyometric training enhances lower limb strength performance and stiffness in elite long jump athletes

With respect to maximal strength gains, the present study found that both ST and ST + PT effectively enhanced maximal strength in elite long jump athletes. This suggests that an 8-week program of either ST or ST + PT provides sufficient stimulus to improve maximal strength in this population. It is well established that strength training loads exceeding 80% of 1RM impose a strong external stimulus on the neuromuscular system and represent an optimal strategy for developing absolute strength. Such adaptations are primarily attributed to neuromuscular changes rather than morphological alterations (Docherty and Sporer, 2000). The strength improvements observed following 8 weeks of ST + PT likely stem from increased motor unit recruitment, higher firing frequency, and improved synchronization. These findings are consistent with previous evidence indicating that strength gains following plyometric interventions are primarily attributable to neuromuscular adaptations rather than hypertrophic changes. In adults, these neural adaptations arise from repeated training stimuli that enhance motor unit activation and coordination.

Lower-limb vertical stiffness is primarily regulated by neuromuscular activation, with changes reflecting adaptations induced by training (Franklin et al., 2003). Vertical stiffness characterizes the overall spring-like behavior of the runner’s lower limbs and reflects the ability to store and release elastic energy in response to vertical ground reaction forces (Brughelli and Cronin, 2008b; Coleman et al., 2012). Within physiological thresholds, higher stiffness allows for greater elastic energy storage during the eccentric phase, thereby enhancing running efficiency by facilitating more effective recoil of elastic tissues and reducing the metabolic cost of muscle work (Fletcher and MacIntosh, 2017). In the present study, ST alone produced only limited improvements in unilateral and bilateral Kvert, whereas ST + PT significantly improved both unilateral (Kvert-L, Kvert-R) and bilateral (Kvert-B) stiffness in male and female athletes. This differential outcome highlights the critical role of plyometric training in optimizing neuromuscular adaptations and improving lower-limb elastic properties. When combined with a well-developed strength base, plyometric training exposes muscles to higher levels of tension than conventional slow-velocity resistance training, applying greater loads over shorter time frames and thereby markedly enhancing lower-limb stiffness.

The observed improvement in vertical stiffness in the ST + PT group is best explained by neuromuscular adaptations specific to plyometric training, which enhances SSC efficiency through exercises such as depth jumps, bounding, and hopping (Mikkola et al., 2007; Pauli et al., 2024). These adaptations involve three principal mechanisms. First, increased motor unit recruitment: muscle stretching activates muscle spindles, eliciting a stretch reflex that enhances motor unit recruitment and/or optimizes rate coding during the subsequent concentric phase (Butler et al., 2003). This augments force output, thereby increasing stiffness and SSC efficiency. Second, reduced inhibitory feedback from Golgi tendon organs (GTOs): in untrained individuals, intense SSC movements may trigger GTO-mediated inhibition, reducing muscle activation and stiffness in the early eccentric phase. Plyometric training attenuates this inhibitory mechanism (disinhibition), thereby preserving SSC function. Third, reduced electromechanical delay (EMD): EMD refers to the latency between neural activation and force production. Plyometric training improves pre-activation of muscles and tendon stiffness, which reduces EMD and enhances the efficiency of force transmission (Kyröläinen et al., 2004).

The finding that male athletes exhibited higher vertical stiffness than females is consistent with previous studies (Hughes and Watkins, 2008; Wang et al., 2015). This difference likely results from males generating greater vertical ground reaction forces and exhibiting smaller vertical displacements of the center of mass. Female athletes in this study may have adopted landing strategies characterized by increased joint range of motion, which reduces ground reaction forces at impact (Hughes and Watkins, 2008). Such strategies may act as protective mechanisms to decrease loading on lower-limb joint structures by allowing greater energy absorption through controlled flexion of the hip, knee, and ankle joints, thereby attenuating impact forces transmitted through the musculoskeletal system.

### 4.2 Combined strength and plyometric training enhances lower limb EEU in elite long jump athletes

The present findings demonstrate that, after 8 weeks of intervention, ST alone produced only limited improvements in unilateral and bilateral vertical stiffness in elite long jump athletes. In contrast, the ST + PT regimen yielded significant increases in both unilateral (Kvert-L, Kvert-R) and bilateral (Kvert-B) stiffness across male and female athletes. This differential improvement can be attributed to the PT component incorporated into the ST + PT program.

As indicated earlier, ST primarily enhances maximal strength, whereas ST + PT promotes improvements in both maximal strength and RFD (Vogt and Hoppeler, 2014). This dual benefit explains the superior gains in EEU observed in the ST + PT group. Elastic energy is stored in muscles and tendons during ground contact and subsequently released during the concentric contraction. Tendons, as the principal storage sites (Kubo et al., 1999; Lichtwark and Wilson, 2007), do not contract independently but transmit tension generated by muscle stiffening. Enhancements in maximal strength and RFD facilitate faster and stronger muscle stiffening, more effective force transmission, and reduced latency during the eccentric-concentric transition, thereby improving EEU.

Following the intervention, maximal strength increased significantly in the ST group, whereas the ST + PT group showed significant gains in both maximal strength and RFD. It is therefore reasonable to infer that the superior EEU improvements observed in ST + PT were largely mediated by RFD enhancements. This interpretation aligns with evidence that, particularly at higher running velocities, the reutilization of elastic energy exceeds the mechanical contribution of muscle contraction, providing the majority of energy required for locomotion. Consistent with this interpretation, the present study found no association between the changes in EEU and the improvements in 1RM squat strength, likely because the slow velocity of the squat descent is suboptimal for elastic energy storage. In contrast, a correlation was observed between EEU and CMJ performance in male athletes, underscoring the critical role of RFD in optimizing EEU.

Biomechanically, elastic tissues in the lower limb store energy during ground contact and release it during push-off. Previous research indicates that this mechanism can contribute 30%–40% of the total energy required for running, with reliance increasing at higher speeds (Damasceno et al., 2015). EEU reduces metabolic energy expenditure and improves running performance (Vogt and Hoppeler, 2014), particularly at high velocities (Mikkola et al., 2007). In the present study, no significant sex differences in EEU were observed.

### 4.3 Combined strength and plyometric training enhances jump performance in elite long jump athletes

Previous research indicates that incorporating diverse strength modalities into athletic training can enhance running and jumping performance. From an energy metabolism perspective, tendons act as primary sites of elastic energy storage but rely on active muscle contractions to realize their functional potential (Kubo et al., 1999; Lichtwark and Wilson, 2007). Enhancements in strength qualities optimize muscle-tendon stiffness and improve the efficiency of elastic energy storage. The current findings demonstrated that EEU improvements were correlated with CMJ performance but not with 1RM squat strength, suggesting that RFD is more critical than maximal strength in high-intensity explosive movements. These results corroborate and extend previous research (Dodd and Alvar, 2007; Mihalik et al., 2008), clarifying the distinct contributions of strength components to athletic performance.

Jumping is a complex, multi-joint movement requiring both high force generation and rapid power output (Fatouros et al., 2000). In the present study, only the ST + PT group demonstrated significant improvements in CMJ height among both male and female athletes following the 8-week intervention. Although modest gains were observed in the ST group, these did not reach statistical significance. This divergence is attributable to the distinct training modalities employed: in ST, CMJ improvements were primarily strength-driven, whereas in ST + PT, improvements stemmed from both maximal strength and the high-power outputs elicited by PT.

PT involves rapid deceleration followed immediately by rapid acceleration in the opposite direction (Fatouros et al., 2000). Such exercises exploit the elastic properties of muscles and connective tissues, allowing energy storage during eccentric loading and its release during concentric contraction. This training approach imposes greater mechanical tension on the muscles compared with traditional slow-velocity resistance training, potentially eliciting post-activation potentiation (PAP), a transient physiological enhancement of muscle strength, contraction velocity, and power output that occurs following maximal or near-maximal intensity exercise, thereby contributing to improved overall power performance (Blazevich and Babault, 2019). Consequently, PT is widely recognized as a highly effective modality for improving muscular power and jump performance (Behm and Sale, 1993; Fatouros et al., 2000). Numerous studies have emphasized the central role of RFD in jump enhancement. For instance (Mihalik et al., 2008), reported that PT increased CMJ height by 5.4%, concluding that ST + PT enhanced neuromuscular function independent of sex, findings consistent with the present study.

These results are also congruent with investigations on PT volume (Chaabene and Negra, 2017), showing that an 8-week plyometric program significantly improved jump performance in male soccer players. Similarly, previous research examined correlations between general and sport-specific jumps in elite male volleyball players, finding significant interrelationships across tests. Collectively, such findings suggest that vertical jump improvements through PT may translate into enhanced competitive performance in elite soccer player (Arnason et al., 2004).

In the present study, sex-based comparisons revealed that the effect size of improvements was significantly greater in male long jump athletes compared to females (P = 0.003). This difference may reflect a combination of factors, including long-term physiological adaptations that are influenced by sex-specific development during adolescence, as well as potential differences in training responses between male and female athletes (Ford et al., 2011; Marceau et al., 2011).

In summary, integrating ST with PT effectively enhances vertical stiffness, EEU, and CMJ performance in elite long jump athletes, with the greatest benefits observed at high running velocities, where the SSC is most fully engaged and improvements in lower-limb stiffness have the strongest impact on performance. These adaptations are directly relevant to long jump performance, as increased stiffness and improved elastic energy utilization facilitate more efficient force transmission during the penultimate and take-off steps, thereby aiding in the maintenance of horizontal velocity and enhancing take-off efficiency. Collectively, these findings provide a robust basis for optimizing training prescriptions in this population.

### 4.4 Limitations

Although the present findings are clinically relevant, several limitations merit attention. First, the study was confined to an elite athlete population; inclusion of a broader demographic spectrum would enhance the generalizability of plyometric training effects on lower limb stiffness in long-jump athletes. Second, the analysis addressed only a discrete component of lower limb stiffness; subsequent investigations should embed these parameters within comprehensive biomechanical examinations of the entire long-jump approach phase to corroborate training efficacy. Third, the training program employed a fixed structure over the intervention period, without explicit consideration of progressive overload or differentiation between fast-SSC and slow-SSC exercises. Future studies could explore varying intensities, SSC modalities, and longer intervention durations to optimize training outcomes and clarify the temporal dynamics of adaptation.

### 4.5 Practical implications

From a practical perspective, long jump coaches are advised to incorporate structured PT into pre-competition training cycles using a progressive overload approach. An effective combined regimen may involve resistance exercises such as squats and split squats performed at 80%–85% 1RM, with gradual increases in load or volume over time, complemented by plyometric drills including 40-cm box drops, hurdle hops, and single-leg hops. Implemented twice per week over 8 weeks, this strategy is likely to yield favorable performance outcomes. The present findings also suggest that improvements in EEU are more strongly associated with enhancements in RFD and stiffness than with maximal strength alone, highlighting the importance of high-power output modalities. Future investigations should expand to athletes with varying training experience, performance levels, and genders, as well as to other jumping disciplines. Longitudinal tracking of different cohorts will clarify the long-term efficacy of plyometric interventions and provide both theoretical and practical guidance for refining training strategies.

## 5 Conclusion

This study demonstrated that an 8-week combined training program, consisting of ST at 80%–85% 1RM and PT, was more effective than ST alone in improving lower limb maximal strength, stiffness, EEU, and jump performance in elite long jump athletes. The combined regimen more effectively optimized neuromuscular function and exercise energy utilization efficiency. Moreover, male athletes exhibited greater training-induced adaptations compared to females. These findings underscore the value of integrating plyometric modalities into conventional strength programs to optimize performance outcomes in elite long jump athletes.

## Data Availability

The raw data supporting the conclusions of this article will be made available by the authors, without undue reservation.
